# Clinical Features of Patients With Hematological Malignancies Treated at the Palliative Care Unit

**DOI:** 10.1089/pmr.2023.0028

**Published:** 2023-09-28

**Authors:** Hiromichi Yamane, Nobuaki Ochi, Ayaka Mimura, Yoko Kosaka, Naruhiko Ichiyama, Tatsuyuki Kawahara, Yasunari Nagasaki, Hidekazu Nakanishi, Nagio Takigawa

**Affiliations:** Department of General Internal Medicine 4, Kawasaki Medical School, Okayama, Japan.

**Keywords:** end-of-life care, palliative care unit, patients with hematological malignancies, survival prediction

## Abstract

**Background:**

In Japan, the number of patients with aggressive hematological malignancies (PHMs) admitted at the palliative care unit (PCU) in their end-of-life (EOL) stage was fewer than that of patients with solid tumors due to several reasons. The assessment of patient characteristics and the methods of survival prediction among PHMs in the EOL stage are warranted.

**Objectives:**

This study aimed to identify the current medical status and the method of survival prediction among PHMs treated at the PCU.

**Setting/Subjects/Measurements:**

We retrospectively analyzed the clinical data of 25 PHMs treated at our PCU between January 2017 and December 2020. The association between survival time and the palliative prognostic score (PAP) and palliative prognostic index (PPI) was analyzed.

**Results:**

The average age of the PHMs was higher than that of patients with lung cancer as a control. The median survival time of the PHMs was shorter than the control group. Most PHMs could not receive standard chemotherapy, and the most common cause of death was disease-related organ failure. Significant associations were observed between the survival time and each PAP/PPI value in patients with malignant lymphoma, but not in those with leukemia.

**Conclusion:**

The PHMs in the PCU had a lower median survival time than the control group. These results were induced by the result of patient selection to avoid treatment-related severe toxicity. The survival prediction using the PAP and PPI was less accurate in patients with leukemia.

## Introduction

The current status of palliative care and the clinical characteristics of patients with aggressive hematological malignancies (PHMs) treated in the palliative care unit (PCU) remain unclear. The American Society of Clinical Oncology has stated that well-planned palliative care and hospice programs in the end-of-life (EOL) period reduced the burden of all patients with malignant tumors and resulted in better clinical outcomes for their caregivers.^1^ Furthermore, palliative care can relieve discomfort, independent of the disease type and prognosis. However, this statement might not apply to PHMs.^2^ PHMs are treated with more chemotherapy, leading to severe adverse events, compared to those with solid tumors, and they frequently experience disease-specific complications, such as fatigue, drowsiness, pain, and dyspnea. These patients and their caregivers often present with psychological distress.^3–5^ A Japanese report has shown that the tumor-related symptoms of PHMs were severe if intensive care treatments, such as frequent blood transfusion and antibiotic usage, were not provided.^5^ Thus, several PCUs in Japan sometimes refuse the admission of PHMs. Based on these results, PHMs in Japan tended to be managed with aggressive chemotherapy regimens at the hematology or clinical oncology division, even in the EOL periods.^4,5^ To improve the quality of life (QOL) and establish a novel standard of EOL care for PHMs, the current medical status of these patients in the real world must be identified and problems encountered in the PCU should be resolved.^6^ Furthermore, an accurate prediction of patient survival is essential for palliative care, and the novel standard of palliative care for PHMs is ready to be established.^6–11^ Hence, the association between survival time and different prognostic indicators must be validated to achieve better clinical outcomes in PHMs and their caregivers.^11,12^

## Materials and Methods

### Study population

The PCU of Kawasaki Medical School General Medical Center was established in December 2016 and has facility attached to the Kawasaki Medical School, located in regional area of Japan. The ward has 16 beds for regular admission and 2 beds for urgent hospitalizations in the PCU. Each year, approximately 80–100 patients with malignant tumors are admitted. To be eligible for PCU admission, patients must meet the following criteria:

1. They must have malignant tumor or acquired immune deficiency syndrome.
2. They should be suffering severely (under 40% in Karnofsky Performance Status as a reference value), and their estimated survival duration should be around one to three months.
3. Patients who wish to improve their activity of daily life (ADL) and QOL rather than extending their survival are eligible, provided they sign the informed consent form for admission. Our institution's PCU always accepts PHMs who require blood transfusions and even patients with severe symptom burden, if they desire.

### Data collection

We recruited 25 PHMs who were admitted at the PCU of Kawasaki Medical School General Medical Center between January 2017 and December 2020 and retrospectively analyzed the clinical data of these patients. To include a sample of patients with solid tumors and a comparison group, 94 patients with lung cancer who were treated in the PCU during the same period were also recruited in this study. Because lung cancer is the most common cause of cancer death and most frequently treated in Japanese PCU, we selected the patients with lung cancer as a control. To calculate the palliative prognostic index (PPI)^11,13^ and palliative prognostic score (PAP) and to report the clinical manifestations, the following data were collected from the medical records by three attending physicians who were the Diplomate, Specialty Board of Palliative Medicine, Japanese Society of Palliative Medicine: age, sex, pathological diagnosis, dose intensity of chemotherapeutic agents, chemotherapy cycle before PCU admission, survival time after PCU admission, direct cause of death, and clinical manifestations.^14^ This study was approved by the Ethics Committee of Kawasaki Medical School (No. 5195-00) and was conducted according to the 1975 Declaration of Helsinki. Informed consent was obtained from the study participants before the study commencement. None of the patients who provided consent was eliminated from this study.

### Survival prediction

#### Palliative prognostic index

As in the study by Morita et al., the PPI was established to predict the survival of terminally ill patients with malignant tumors, including small amounts of hematological malignant tumors, and was calculated using the following variables: performance status, oral intake, edema, dyspnea at rest, and delirium.^13^ These variables are associated with clinical symptoms and can be collected easily from the medical records. If a PPI >6 was adopted as the cutoff, the sensitivity and specificity of predicting less than three weeks of survival were 80% and 85%, respectively.^13^

#### Palliative prognostic score

As in the study by Pirovano et al., the PAP was established for terminally ill patients with solid tumors and was defined by assessing the following variables: dyspnea, anorexia, Karnofsky performance status, total leukocyte count, lymphocyte percentage, and clinician-estimated survival time.^14^ The PAP ranges from 0 to 17.5. Based on the PAP, the patients were classified into three risk groups according to the probability of 30-day survival and expected survival time: group A (score: 0–5.5; probability of 30-day survival: >70%, 95% confidence interval [CI] of survival time: 67–87 days), group B (score: 6–11; probability of 30-day survival: 30%–70%, 95% CI of survival time: 28–39 days), and group C (score: 11.5–17.5; probability of 30-day survival: <30%, 95% CI of survival time: 11–18 days).^14^

### Statistical analysis

PPI and PAP were calculated on the first day of PCU admission. Overall survival time was calculated from the admission day to the patient's death. If applicable, Student's *t* test or the Mann–Whitney U test was used to assess differences in each variable between the subject groups, and the chi-square test, or Fisher's exact test, was used to assess positive quantitative differences between the subject groups. Survival was examined using the Kaplan–Meier method, and the difference was evaluated using the log-rank test. Pearson's correlation coefficient of the association between the survival time and PPI or PAP was calculated using STATA (Light Stone Corp., Tokyo, Japan). All *p*-values corresponded to the two-sided tests, and *p*-values <0.05 were used to denote statistical significance.

## Results

### Characteristics of the PHMs treated in the PCU

**FIG. 1.**
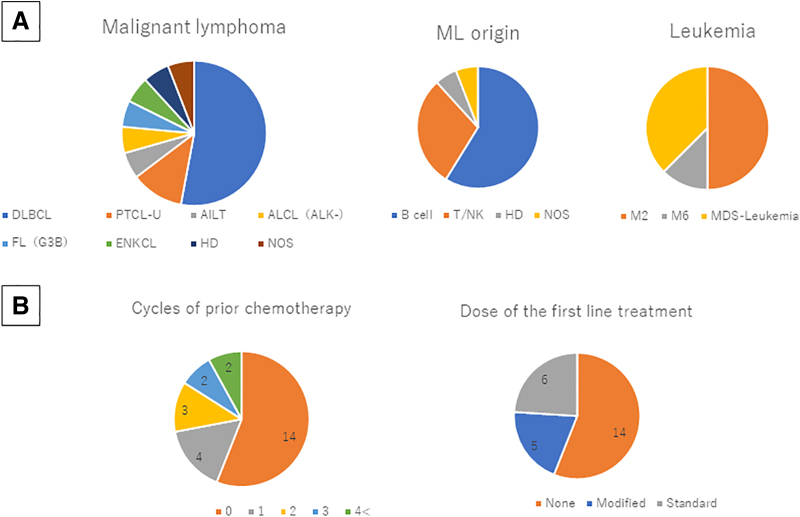
**(A)** Pathological diagnosis. The incidence of malignant lymphoma was similar to that in a Japanese nationwide survey, and significant deviation was not observed in each disease entity. **(B)** Initial treatment and extent of therapy. Approximately 56% of patients with hematological malignancy (*n* = 14) were not treated with anticancer agents. Only 28% of patients received salvage chemotherapy, and 16% of patients received more than three regimens. Of 11 patients treated with anticancer agents, 6 received the standard regimen as an initial treatment. However, the residual five patients were treated with modified or dose-reductive treatment regimen. AILT, angioimmunoblastic T cell lymphoma; ALCL, anaplastic large cell lymphoma; DLBCL, diffuse large B cell lymphoma; ENKCL, extranodal nasal-type natural killer/T cell lymphoma and NK-cell leukemia; FL (G3B), follicular lymphoma grade 3B; HD, Hodgkin lymphoma; M2, acute differentiated myeloblastic leukemia (acute myeloid leukemia M2); M6, erythroleukemia (acute myeloid leukemia M6); MDS-leukemia, acute myeloid leukemia transformed from myelodysplastic syndrome; ML, malignant lymphoma; NOS, malignant lymphoma not otherwise specified; PTCL-U, peripheral T cell lymphoma unspecified; T/NK, natural killer/T cell.

Table 1 shows the characteristics of the PHMs treated in the PCU. The group comprised 25 patients (13 men and 12 women), aged 62–95 years (mean ± standard deviation: 84.4 ± 7.64 years). In total, 17 patients (68% of all patients) had malignant lymphoma (ML), and eight (32% of all patients) presented with acute myeloid leukemia (AML). The incidence of ML was similar to that in a Japanese nationwide survey, and a significant deviation was not observed in each disease entity of ML (Table 1; Fig. 1A). Among the eight patients with AML, four (50% of AML) were diagnosed with AML transformed from myelodysplastic syndrome based on data from the medical records and the findings of optical microscopic examination and bone marrow chromosome analysis. Four patients (50% of AML) were diagnosed with *de novo* AML. Among the four patients with *de novo* AML, three (75%) were diagnosed with acute differentiated myeloblastic leukemia (AML-M2) and one (25% of *de novo* AML) with erythroleukemia (AML-M6) based on the optical microscopic findings of peripheral blood smear using the French–American–British classification. The patient's performance status was extremely poor. Hence, the World Health Organization classification could not be determined (Fig. 1A). The average age was 84.04 ± 7.8 years for the PHMs and 75.31 ± 9.76 years for the patients with lung cancer. On average, the PHMs were significantly older than patients with lung cancer (*p* < 0.01) (Supplementary Fig. S1A).

**Table 1.** Patients Characteristics (Hematological Malignancies; *n* = 25)

	Patients ***n*** (%)
Age, mean ± SD (range)	84.4 ± 7.64 (62–95)
Male	13 (52)
Disease	
Lymphoma	17 (68)
NHL	16 (64)
Hodgkin	1 (4)
Leukemia	8 (32)
*De novo*	5 (20)
MDS base	3 (12)
Prior chemotherapy	
0	14 (56)
1	4 (16)
2	3 (12)
3	2 (8)
4 or less	2 (8)
Dose of the first-line treatment	
Modified	5 (20)
Standard	6 (24)
None	14 (56)
Pattern of death	
Tumor death	20 (80)
Infection	3 (12)
Exacerbation of complications	2 (8)
Transfusion	7 (28)

MDS, myelodysplastic syndrome; NHL, non-Hodgkin's lymphoma; SD, standard deviation.

### Initial treatment of the PHMs before PCU admission

Approximately 56% of the PHMs treated in the PCU (*n* = 14) did not receive an anticancer agent because of old age, poor performance status, and/or complicated organ dysfunction. Approximately 28% of these patients (*n* = 7) received salvage chemotherapy, of which only 16% (*n* = 4) received more than three regimens. Among the 11 patients (44% of all patients) with hematological malignancies treated with anticancer agents, 6 (24% of all patients) received the standard regimen as the initial treatment. However, five residual patients (20% of all patients) were treated with schedule-modified or dose reductive treatment regimens. The clinical outcomes of these patients treated with nonstandard treatments were insufficient (Table 1; Fig. 1B; Supplementary Fig. S1B).

### Direct cause of death

**FIG. 2.**
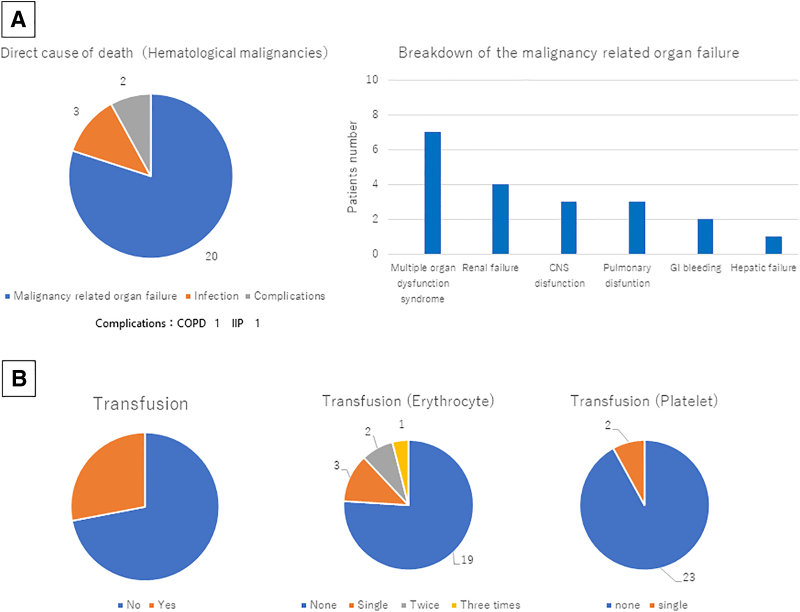
**(A)** Direct cause of death. Approximately 80% of patients died due to disease-related organ failure or organ failure caused by treatment-related adverse events, 12% due to infection, and 8% due to complications. Among patients who died due to malignancy-related organ failure, seven presented with multiple organ dysfunction syndrome, four with renal failure, and three with cerebral nervous system dysfunction and pulmonary dysfunction. Two patients died owing to hypotension caused by GI bleeding. One patient died due to hepatic failure and hepatosplenomegaly. **(B)** Blood transfusion in patients with hematological malignancies. Among 25 patients with hematological malignancies, 7 required blood transfusion, and 6 patients needed erythrocyte transfusion. Two patients with GI bleeding required single platelet transfusion. One patient who needed platelet transfusion also required erythrocyte transfusion because of dyspnea due to anemia caused by GI bleeding. CNS, central nervous system; COPD, chronic obstructive pulmonary disease; GI, gastrointestinal; IIP, idiopathic interstitial pneumonia.

Next, we validated the direct cause of death among the PHMs. Approximately 80% of the patients (*n* = 20) died because of organ failure caused by the clinical course of hematological malignancies or organ failure caused by treatment-related adverse events. Approximately 12% of the patients (*n* = 3) died because of infection due to disease-related immunodeficiency, such as pneumonia, adult respiratory distress syndrome caused by pneumonia, and acute obstructive suppurative cholangitis. Approximately 8% of the patients (*n* = 2) died of pulmonary complications, such as acute exacerbation of chronic obstructive pulmonary disease and idiopathic pulmonary fibrosis. However, these acute exacerbations may have been caused by disease-related immunological disorders. Among the 20 patients (80% of all patients) who died of malignancy-related organ failure, 7 (28% of all patients) presented with multiple organ dysfunction syndromes, which are characterized by two or more organ failure progressing simultaneously; 4 (16% of all patients) presented with renal failure; and 3 (12% of all patients) presented with cerebral nervous system dysfunction and pulmonary dysfunction. Two PHMs (8% of all patients) died of hypotension caused by gastrointestinal bleeding, and these patients had severe thrombocytopenia for which frequent transfusion was necessary. The other patient (4% of all patients) died of hepatic dysfunction and hepatosplenomegaly, which may have been caused by tumor infiltration to reticuloendothelial organs (Fig. 2A).

### Blood transfusion

In this study, seven patients with central nervous system dysfunction or pulmonary dysfunction caused by tumor involvement to meninx or lung, respectively, required blood transfusion to control symptoms, such as dizziness, dyspnea, fatigue, and bleeding. Six patients required erythrocyte transfusion to control dyspnea and intractable fatigue caused by heart failure, owing to severe anemia. One patient required erythrocyte transfusion thrice. Two patients needed erythrocyte transfusion twice, and three patients required single erythrocyte transfusion during the EOL period in the PCU. Two patients with gastrointestinal bleeding needed platelet transfusion. One of the patients who needed platelet transfusion required erythrocyte transfusion thrice to control suffering (Fig. 2B).

### Survival time after PCU admission

Next, we compared the survival time of the patients with each disease in this study. The average survival time of the PHMs and those with lung cancer admitted to the PCU were 23.44 ± 23.24 and 41.37 ± 52.6 days, respectively. The PHMs had a significantly lower average survival time and survival time in the PCU than those with lung cancer (*p* < 0.05) (Supplementary Fig. S1B). The average survival times of patients with AML and ML were 27.75 ± 28.47 and 20.29 ± 19.27 days, respectively. No significant difference in the average survival time was observed between patients with AML and those with ML admitted to the PCU (*p* = 0.468) (Supplementary Fig. S1C). The PHMs had a shorter survival time than patients with lung cancer.

### Analysis of survival predictors

**FIG. 3.**
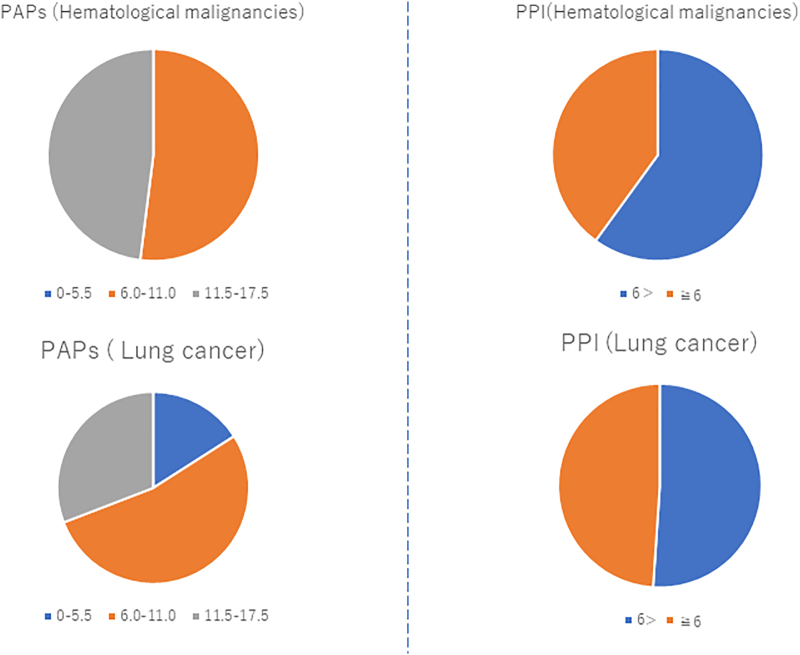
PPI and PAP of patients with hematological malignancies. None of the patients with hematological malignancies who had a PAP of <5.5 might survive less than two or three months. PAP, palliative prognostic score; PPI, palliative prognostic index.

**FIG. 4.**
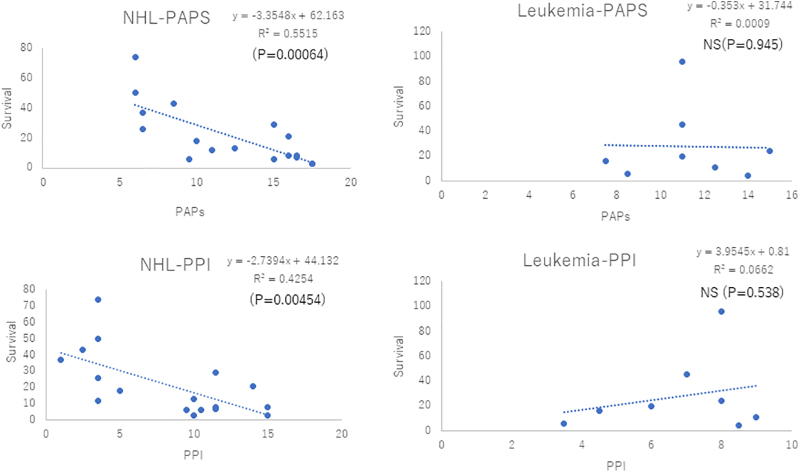
Association between PPI/PAP and survival time among hematological malignancies. There was no association between survival predictors and survival time in patients with AML. PPI and PAP may be useful for survival prediction in patients with NHL. AML, acute myeloid leukemia; NHL, non-Hodgkin's lymphoma.

The PAP and PPI were evaluated, and the association between the survival time and PAP/PPI was analyzed. The average PPI and PAP of the 25 PHMs were 7.82 ± 3.97 and 11.88 ± 3.77, respectively. The average PPI and PAP of the 94 patients with lung cancer were 5.71 ± 3.13 and 9.37 ± 3.63, respectively. The PHMs had higher PPI (*p* < 0.05) and PAP (*p* < 0.01) than those with lung cancer. However, no significant differences in the PPI and PAP were observed between patients with AML and those with ML (*p* = 0.406 vs. 0.623) (Supplementary Fig. S2). Then, the details of survival prediction were analyzed using the PPI or PAP. The PPI of ∼40% of the PHMs was >6.0. Moreover, the PPI of ∼48.9% of patients with lung cancer was >6.0. The proportion of patients with lung cancer and those with hematological malignancies with a PPI >6.0 was almost similar, and the value did not significantly differ between the PHMs and those with lung cancer (*p* = 0.426) (Fig. 3). Next, the PAP was examined. The examination showed that 52% of the PHMs and 53% of those with lung cancer had belonged to group B, which indicated a 30%–70% probability of 30-day survival. Thus, no significant difference was observed between the two groups. However, the incidence of group A, which indicated >70% probability of 30-day survival, was quite different between the two groups. Although no PHMs belonged to group A, only 16% of those with lung cancer were classified into group A. A significant difference was observed between the two groups (*p* < 0.05) (Fig. 3). However, to confirm this result, the correlation between the actual survival time and the PAP or PPI was assessed. The PAP and PPI were inversely correlated with the survival time at the PCU among the PHMs and those with lung cancer. These correlations may be more evident in patients with lung cancer than in PHMs after comparing the statistical parameters of each analysis (Supplementary Fig. S3). Subsequently, we validated the correlation between the survival time and PAP/PPI among the 25 PHMs. In 17 patients with ML, both the PAP and PPI were inversely correlated with the survival time (*p* < 0.01) (Fig. 4). In contrast, we could not identify any correlation between the survival time in the PCU and the PAP/PPI among the eight patients with AML. Hence, the PPI and PAP may not be accurate for survival prediction in patients with AML but accurate in patients with non-Hodgkin's lymphoma (NHL) (Fig. 4).

## Discussion

In this study, the characteristics and prognosis of the PHMs treated at our PCU were evaluated. Next, the correlation between the actual survival time and prognostic indicators was investigated.

On average, the PHMs were significantly older than patients with lung cancer, and the survival time of the PHMs was significantly shorter than that of patients with lung cancer. These phenomena may be explained by the aggressive course of hematological malignancies and the direct cause of death, which was common in this study. Approximately 80% of the patients died of malignancy-related organ failure or organ failure due to treatment-related adverse events, and 12% of the patients died of infection. Over 90% of the candidates died of tumor-related organ failure and/or infection caused by tumor-related immunodeficiency. In a Japanese cancer survey, patients with leukemia and ML aged >70 years had a significant increase in age-specific mortality risk.^15^ Stated differently, PHMs are at a higher risk of mortality after ∼70 years of age than those with solid tumors due to their unique clinical course. Furthermore, other studies in Europe and eastern Asia had similar results.^16,17^ There might be an evident correlation between age and prognosis.^16–19^ Hence, old PHMs who were treated in the PCU were more likely to have a poor survival. Approximately 50% of the PHMs in the PCU did not receive anticancer agents, only 28% of whom had salvage chemotherapy. We believe that previous treatment regimens could have resulted in the frequent need for blood transfusion in the PCU. Some reports had shown that PHMs were more likely to have a high frequency of blood transfusions fundamentally, and blood transfusions based on detailed medical examination results and medical guidelines were effective in controlling patient symptoms.^18,20,21^

Unfortunately, many PCU physicians in Japan tend to refuse the admission of PHMs because of the difficulty associated with blood transfusion and/or symptom management in case of disease progression. Our aim is to highlight the present state of PHMs at PCU in our hospital and to encourage Japanese medical staff to be more proactive in treating PHMs at PCU to effectively manage their suffering. Through clinical practice, we have observed that blood transfusions are not always considered standard care for people with PHMs in Japanese PCU. Previous research reported many PHMs wanted to continue to receive blood transfusions for symptom control of anemia near the EOL.^22,23^ This was seen in our study with one-third of patients continuing blood transfusions, likely for symptom control.

The PHMs in this experiment could not receive standard chemotherapy as the initial treatment.

Although blood transfusion is not always included in the treatment modality during the EOL period in Japan, PHMs wanted to receive adequate palliative care in the PCU. These patients were more likely to have severe tumor-related symptoms, such as dyspnea caused by anemia. We believe that this was the main reason that almost one-third of the patients in our PCU received blood transfusions to control symptoms. Because two-thirds of PHMs did not require a blood transfusion even if without chemotherapy to control tumor burden, it seemed to be absurd to refuse the admission of PHMs in most Japanese PCUs. Next, this study showed an association between the direct cause of death and the survival time in the PCU among the PHMs. Analyzing the direct cause of death in the PCU is essential to identify the methods of EOL care and the outcome of palliative care. A report has revealed that the main causes of death among patients with AML were organ failure; infections, such as pneumonia, intracranial bleeding due to disease-related bleeding tendency, heart failure, hepatic dysfunction, and renal failure.^24^ Another report has also revealed that patients with NHL who presented with severe symptoms were more likely to choose a palliative care program if their residual survival time was shorter than three weeks.^25^ PHMs might be more likely to be admitted to the PCU during the EOL period, and patients with a poor prognosis had severe symptoms and were easily to end their lives. This presumption may support our findings that >90% of PHMs died of tumor-related organ failure and/or infection caused by tumor-related immunodeficiency and that their survival times were extremely short in the PCU.

As another hypothesis from a different viewpoint, the possibility that the patients who were analyzed in this experiment had inadequate feasibility to receive standard treatment because of poor performance status and age-related organ dysfunction must be discussed. These patients tend to be presented to the regional palliative care program or be presented to the PCU at the cancer center without EOL discussion or excessive chemotherapy because of only a few treatment alternatives. If the results obtained in this experiment reflect these backgrounds, our data seemed reasonable enough.

The accurate prediction of patient survival is essential for palliative care. Some studies have revealed the efficacy of different tools used for survival prediction.^11–13,26,27^ In this retrospective study, the PPI and PAP were selected because of the expedience of data acquisition. The PPI and PAP were inversely correlated with the survival time in patients with ML. However, we could not find any correlation between them and patient survival in AML. A Japanese study has confirmed the validity and usefulness of the PAP when used in survival prediction. The results showed that the PAP was useful in terminally ill PHMs as in those with solid tumors.^28^ Based on these findings, the accuracy of survival prediction using the PAP was higher in patients with ML. Unexpectedly, no correlation was observed between both the PPI and PAP and survival among patients with AML in our study. Although the cause remains unclear, the usefulness of both the PAP and PPI when used as survival predictors is evident in NHL only, not in AML. Nevertheless, a further study with a larger sample size should be conducted to validate our results and to establish a novel standard of palliative care in PHMs.

## Conclusion

The average age of PHMs who received EOL care at a Japanese PCU was high, and the treatment dose intensity of first-line chemotherapy was extremely low. Furthermore, the survival time in PHMs was shorter than that in the control group. In patients with AML, survival prediction using the PAP and PPI was insufficient. Although there was some selection bias in this study, the accomplishment of rapid improvement in the QOL of PHMs seems difficult. Many Japanese PHMs should be adequately treated at PCU to manage their suffering. The establishment of a new standard care for PHMs is warranted.

## Supplementary Material

Supplemental data

Supplemental data

Supplemental data

## Abbreviations Used

AILT: angioimmunoblastic T cell Lymphoma
ALCL: anaplastic large cell lymphoma
AML: acute myeloid leukemia
CI: confidence interval
CNS: central nervous system
COPD: chronic obstructive pulmonary disease
DLBCL: diffuse large B cell lymphoma
ENKCL: extranodal nasal-type natural killer/T cell lymphoma and NK-cell leukemia
EOL: end of life
FL (G3B): follicular lymphoma grade 3B
GI: gastrointestinal
HD: Hodgkin lymphoma
IIP: idiopathic interstitial pneumonia
M2: acute differentiated myeloblastic leukemia (acute myeloid leukemia M2)
M6: erythroleukemia (acute myeloid leukemia M6)
MDS-leukemia: acute myeloid leukemia transformed from myelodysplastic syndrome
ML: malignant lymphoma
NHL: non-Hodgkin's lymphoma
NOS: malignant lymphoma not otherwise specified
PAP: palliative prognostic index
PCU: palliative care unit
PHM: patients with aggressive hematological malignancies
PPI: palliative prognostic score
PTCL-U: peripheral T cell lymphoma unspecified
QOL: quality of life
SD: standard deviation
T/NK: natural killer/T cell
