# Supplementary material for: Clinical Features of Patients With Hematological Malignancies Treated at the Palliative Care Unit
Source: Palliat Med Rep. 2023 Sep 28;4(1):278–87. doi: 10.1089/pmr.2023.0028 (PMC10541919; doi:10.1089/pmr.2023.0028)

**Supplementary Figure 1**

**A: Patient’s age.**

Patients with hematological malignancies were older than those with lung cancer (P < 0.0001). Hematological malignancy: 84.04 +/- 7.80 years old. Lung cancer: 75.31 +/- 9.76 years old.

**B: Survival after palliative care unit (PCU) admission between patients with hematological malignancies and those with lung cancer.**

Patients with hematological malignancies admitted at the PCU had a shorter survival time than those with lung cancer (P = 0.014). Hematological malignancies: 23.44 +/- 23.24 days. Lung cancer: 41.37 +/- 52.6 days.

**C: Survival after PCU admission between patients with acute myeloid leukemia (AML) and those with malignant lymphoma (ML).**

There was no significant difference in terms of survival time after PCU admission between patients with AML and those with ML (P = 0.468).


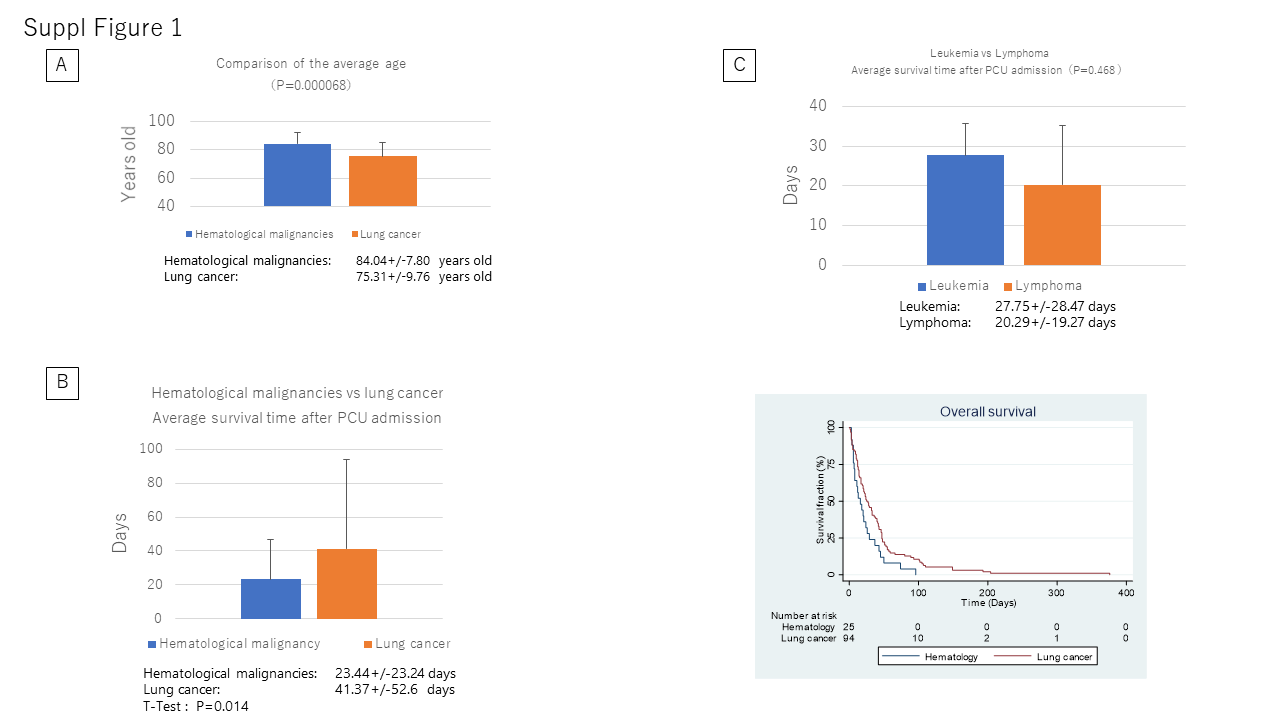

Supplement: Supplemental data [file Suppl_FigS1.docx]
