# Supplementary material for: Clinical Features of Patients With Hematological Malignancies Treated at the Palliative Care Unit
Source: Palliat Med Rep. 2023 Sep 28;4(1):278–87. doi: 10.1089/pmr.2023.0028 (PMC10541919; doi:10.1089/pmr.2023.0028)

**Supplementary Figure 2**

**Palliative Prognostic Score (PAP) and Palliative Prognostic Index (PPI) between patients with AML and those with ML.**

Patients with hematological malignancies had a higher average PPI and PAP than those with lung cancer. However, among patients with hematological malignancies, no significant difference was observed in terms of the average PAP and PPI.


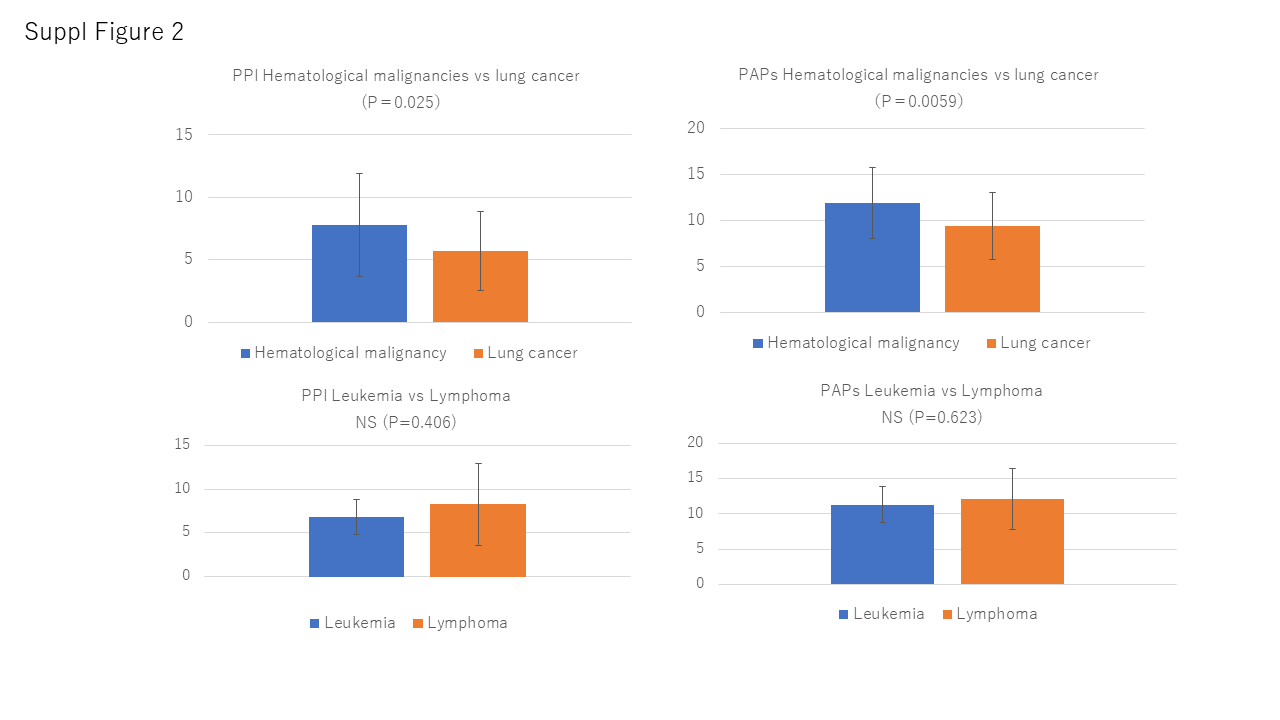

Supplement: Supplemental data [file Suppl_FigS2.docx]
