# Supplementary material for: Clinical Features of Patients With Hematological Malignancies Treated at the Palliative Care Unit
Source: Palliat Med Rep. 2023 Sep 28;4(1):278–87. doi: 10.1089/pmr.2023.0028 (PMC10541919; doi:10.1089/pmr.2023.0028)

**Supplementary Figure 3**

**The survival predictors**

The survival predictors were inversely correlated with the survival time in patients with hematological malignancies and those with lung cancer.


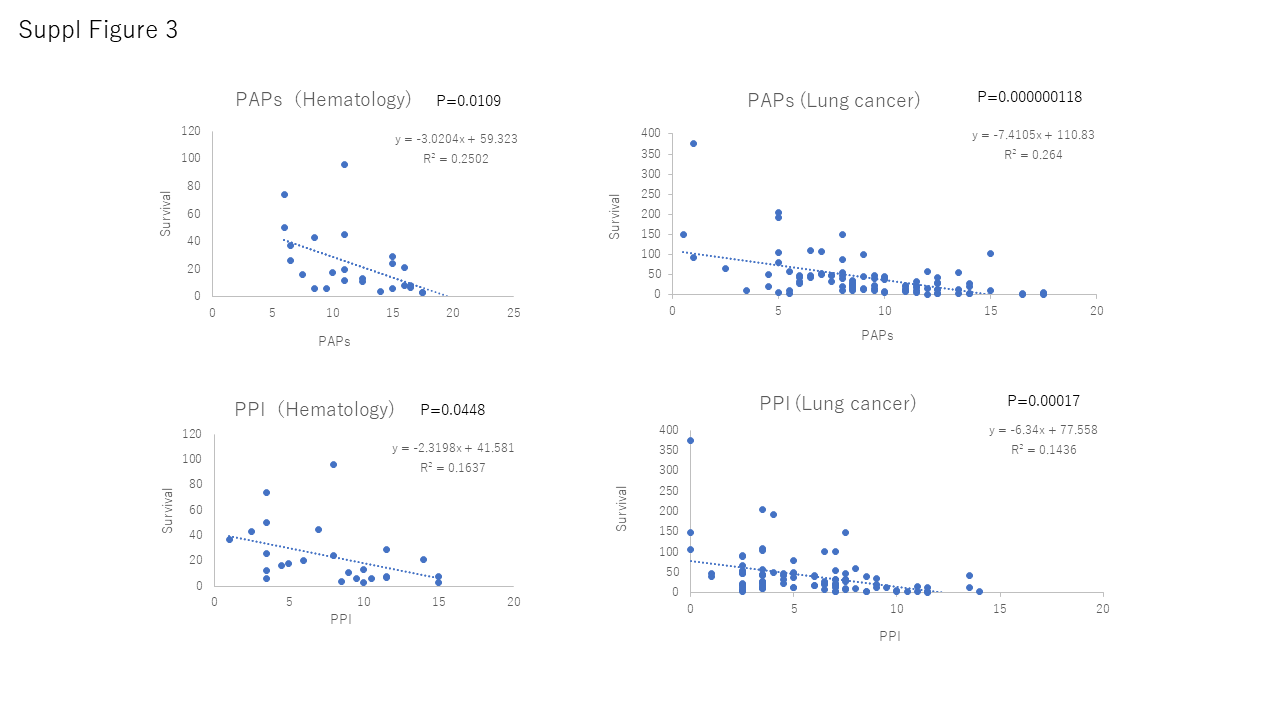

Supplement: Supplemental data [file Suppl_FigS3.docx]
